# Single‐Cell RNA Sequencing Analysis Reveals Exercise‐Induced Transcriptional Dynamics in Half‐Marathon Runners

**DOI:** 10.1111/sms.70018

**Published:** 2025-01-16

**Authors:** Laura Veschetti, Cristina Patuzzo, Mirko Treccani, Lucas Moron Dalla Tor, Michela Deiana, Samuele Cheri, Francesca Griggio, Giuseppe Lippi, Federico Schena, Daniele De Santis, Luca Dalle Carbonare, Cantor Tarperi, Elisabetta Trabetti, Maria Teresa Valenti, Giovanni Malerba

**Affiliations:** ^1^ Infections and Cystic Fibrosis Unit, Division of Immunology, Transplantation and Infectious Diseases IRCCS San Raffaele Scientific Institute Milano Italy; ^2^ Vita‐Salute San Raffaele University Milano Italy; ^3^ Department of Neurosciences, Biomedicine and Movement Sciences University of Verona Verona Italy; ^4^ Department of Surgical Sciences, Dentistry, Gynaecology and Paediatrics University of Verona Verona Italy; ^5^ Department of Medicine and Surgery University of Parma Parma Italy; ^6^ Department of Infectious‐Tropical Diseases and Microbiology IRCCS Sacro Cuore Don Calabria Hospital Verona Italy; ^7^ Department of Biomedical Sciences University of Cagliari Cagliari Italy; ^8^ Centro Piattaforme Tecnologiche University of Verona Verona Italy; ^9^ Department of Engineering for Innovation Medicine University of Verona Verona Italy

**Keywords:** immune modulation, running, single‐cell RNA sequencing

## Abstract

Previous studies in sports science suggested that regular exercise has a positive impact on human health. However, the effects of endurance sports and their underlying mechanisms are still not completely understood. One of the main debates regards the modulation of immune dynamics in high‐intensity exercise. As part of the “Run 4 Science” project in Verona, Italy, we conducted a single‐cell RNA sequencing analysis on half‐marathon amateur runners to investigate the transcriptional dynamics of peripheral blood mononuclear cells following endurance exercise. Blood samples were collected from four participants before and after running a half‐marathon to carry out a comprehensive transcriptomic analysis of immune cells at the single‐cell level. Our analysis revealed significant alterations in the transcriptional profiles following endurance physical exercise. Modulations in myeloid cells suggested the activation of stress response (6 related pathways, *p* < 0.04) and pathways related to viral processes (4 related pathways, *p* < 0.03), while in lymphoid cells they hinted to a shift towards immune activation (24 related pathways, *p* < 0.01). Additionally, transcriptional changes in platelets point to an activation of the coagulation process (5 related pathways, *p* < 0.005). Single‐cell data was also analyzed following a pseudo‐bulk approach (i.e., simulating a bulk RNAseq experiment) to gain further biological insights. Our findings suggest that a pseudo‐bulk analysis could offer complementary findings to classical single‐cell analysis methods and demonstrate that endurance physical exercise, such as running a half‐marathon, induces substantial changes in the transcriptional dynamics of immune cells. These insights contribute to a better understanding of the immune modulation mediated by endurance exercise and may inform future training routines or nutritional guidelines based on individual gene expression levels.

## 1 Introduction

In the last 20 years, researchers have investigated the impact of practicing regular exercise on human health [1, 2, 3] and the processes underlying exercise in the cardiovascular [4], musculoskeletal [5] and immune systems [6]. The “Run 4 Science” project was initiated in 2014 in Verona (Italy) to address scientific questions regarding endurance sports and high‐intensity activity in professional and amateur athletes [7, 8]. In this context, previous research investigated the effect of physical exercise pointing out related benefits in health and chronic degenerative conditions [9]. In particular, the differentiation of mesenchymal circulating progenitor cells was explored, outlining their role in osteogenic and chondrogenic differentiation and their autophagic response to intense activity.

Even though regular exercise has been suggested to be beneficial for human health, the effect of practising endurance activities still needs to be elucidated. Interestingly, evidence has been reported describing immune dynamics in high‐intensity exercise, giving rise to two hypotheses [10, 11]. The “open window” hypothesis postulates a short‐term immunosuppression action in the hours immediately after intense physical activity [12]. Whereas, the “acute stress‐induced immunoenhancement” hypothesis is that exercise does not suppress the immune system, but that immune cells temporarily redistribute themselves in peripheral tissues before returning to circulation [13].

Recent research explored the immune transcriptional dynamics during physical exercise, focusing on the characterization of cell functions [14] and the modulation of gene expression levels [15]. The great majority of available research data come from bulk RNAseq experiments, which measures the average expression across a population of cells and overlooks cellular heterogeneity. In recent years, single‐cell RNA sequencing (scRNAseq) approaches have been developed and it is now possible to characterize RNA transcriptional profiles at the single cell level, thus gaining insights on cell type‐specific modulations [16]. In the context of the “Run 4 Science” project, we performed a scRNAseq experiment on half‐marathon amateur runners to shed light on endurance exercise‐mediated immune modulation.

## 2 Materials and Methods

### 2.1 Samples Collection and Peripheral Blood Mononuclear Cells (PBMCs) Isolation

Four age‐matched amateur runners (two males and two females, 29 ± 5 years) were enrolled during the annual sports event called “Run 4 Science”, a 21.1 km half marathon held in Verona (Italy) in April 2019. Amateur runners were randomly selected from a sample of approximately 100 individuals present at the event; the only constraints were to select self‐reportedly healthy males and females within the same age range. Written informed consent was obtained from all participants, and the study was approved by the ethical committee of Azienda Ospedaliera Universitaria Integrata of Verona, Italy (number 1538; Dec. 3, 2012; local ethical committee of Azienda Ospedaliera Integrata di Verona). The study design and methods comply with the Declaration of Helsinki. Peripheral blood was collected by venipuncture before the run (pre run) and immediately after (post run). Pre run blood sampling occurred 90–60 min before the scheduled start time of 9 a.m., whereas post run blood sampling was performed 15–30 min after arrival, approximately 3 h after the start time (11:45–12:30 a.m.). In both cases, samples were kept at 10°C–15°C, and within a maximum of 2 h after sampling, (PBMCs) were purified by density gradient centrifugation with Ficoll‐Paque (GE Healthcare). PBMCs were then resuspended in 1X PBS—0.1% filtered BSA and left at 5°C–10°C for up to 1 h before further processing them for sequencing library preparation. The final libraries were amplified and sequenced on an Illumina platform the following day.

### 2.2 Single‐Cell RNA Sequencing (scRNAseq) Library Preparation and Sequencing

RNA‐Seq libraries from single PBMCs were prepared using Bio‐Rad ddSEQ Single‐Cell Isolator and reagents provided in the Illumina Bio‐Rad SureCell WTA 3' Library Prep Kit. Briefly, single PBMCs were individually partitioned into droplets using Bio‐Rad ddSEQ Single‐Cell Isolator. After cell lysis, reverse transcription was performed in each droplet, and mRNA transcripts were barcoded. Then, droplets were disrupted, and second‐strand synthesis took place with pooled barcoded cDNA. Primer binding sites were added by tagmentation (Nextera SureCell transposome) for subsequent indexing. Final libraries were amplified by PCR and purified. After accurate quantification, libraries were ready for sequencing on the Illumina NextSeq 500 platform. Samples from one amateur runner did not produce libraries of sufficient quality and were thus excluded from the sequencing experiment.

### 2.3 Analysis of scRNAseq Data

Raw FASTQ files were processed using ddSeeker version 1.2.1 [17] to extract cellular and molecular identifiers and generate unmapped BAM (uBAM) files. STARsolo version 2.7.11a [18] was used to align uBAM files against the *Homo sapiens* GRCh38 genome and to perform transcript quantification. Count matrices were then processed using Seurat R package version 4.9.9 [19] in an R 4.3.1 [20] environment. Low‐quality cells were removed if unique molecular identifiers (UMIs) counts were fewer than 200, gene counts were fewer than 200, or more than 20% of UMIs derived from the mitochondrial genome. All remaining cells were considered high‐quality cells. Samples from one amateur runner did not have sufficient high‐quality cells and were thus excluded from further analyses. Additionally, all genes not detected in at least 10 single cells in the whole dataset were discarded. The batch effect, effect of mitochondrial expression, and effect of the cell cycle were explored as sources of confounding variation, and the generated datasets were combined using the ‘IntegrateData’ function of Seurat following the SCTransform workflow [21, 22] for data normalization.

### 2.4 Clustering and Cell Subtype Annotation

After normalization and scaling, which allow samples to become comparable, principal component analysis was used for data dimensional reduction. To select the number of principal components (PCs) to use for clustering, we selected the point where the PCs only contribute 5% of the standard deviation and the PCs cumulatively contribute 90% of the standard deviation. The first 15 PCs and a resolution parameter of 0.8 were used in the Seurat FindClusters function. Cell‐type annotation was performed using SCType [23] and the SCType Cell Marker Database for “Immune system”.

### 2.5 Differential Expression Analysis and Gene Set Enrichment Analysis

Gene‐expression changes between pre and post run samples were evaluated individually in each annotated cell cluster following a pseudo‐bulk RNAseq approach using DESeq2 package version 1.40.2 [24] and a paired design. The package allows the estimation of variance–mean dependence in count data from high‐throughput sequencing assays and tests for differential expression based on a model using the negative binomial distribution. The pre run timepoint was used as the base level. Genes showing a |log2FoldChange| > 0.58 (corresponding to a 50% modulation in gene expression) and adjusted *p* < 0.05 were considered as differentially expressed. Gene set enrichment analysis (GSEA) was conducted using clusterProfiler package version 4.8.3 [25] and the gene set annotation for *Homo sapiens* available through the org.Hs.eg.db version 3.17.0. A comprehensive list of all expressed genes ranked by their log2FoldChange was used as input, gene sets with a minimal size of 3 and a maximal size of 800 genes were considered for the analysis, and 100 000 permutations were performed. Gene sets showing an adjusted *p* < 0.05 (Benjamini‐Hochberg correction) were considered as modulated. All plots were generated with ggplot2 version 3.4.4 [26].

### 2.6 Cluster Differential Abundance Analysis

Cell abundance changes were evaluated in each annotated cell cluster with the edgeR package version 3.42.4 [27]. Cell counts normalized by the total number of cells in each sample were used as input, and the glmQLFTest function was used to test differences in the proportion of cells in each cluster. The pre run timepoint was used as the base level. Results showing an adjusted *p* < 0.05 and an FDR < 0.05 were considered statistically significant.

### 2.7 Pseudo‐Bulk Analysis

Differential expression and gene set enrichment analyses were also carried out, simulating a bulk RNAseq experiment starting from the scRNAseq data and considering all cells as belonging to a unique cluster. The methodology and parameters used in the analyses are the same as the ones described for scRNAseq data analysis.

## 3 Results

**FIGURE 1:**
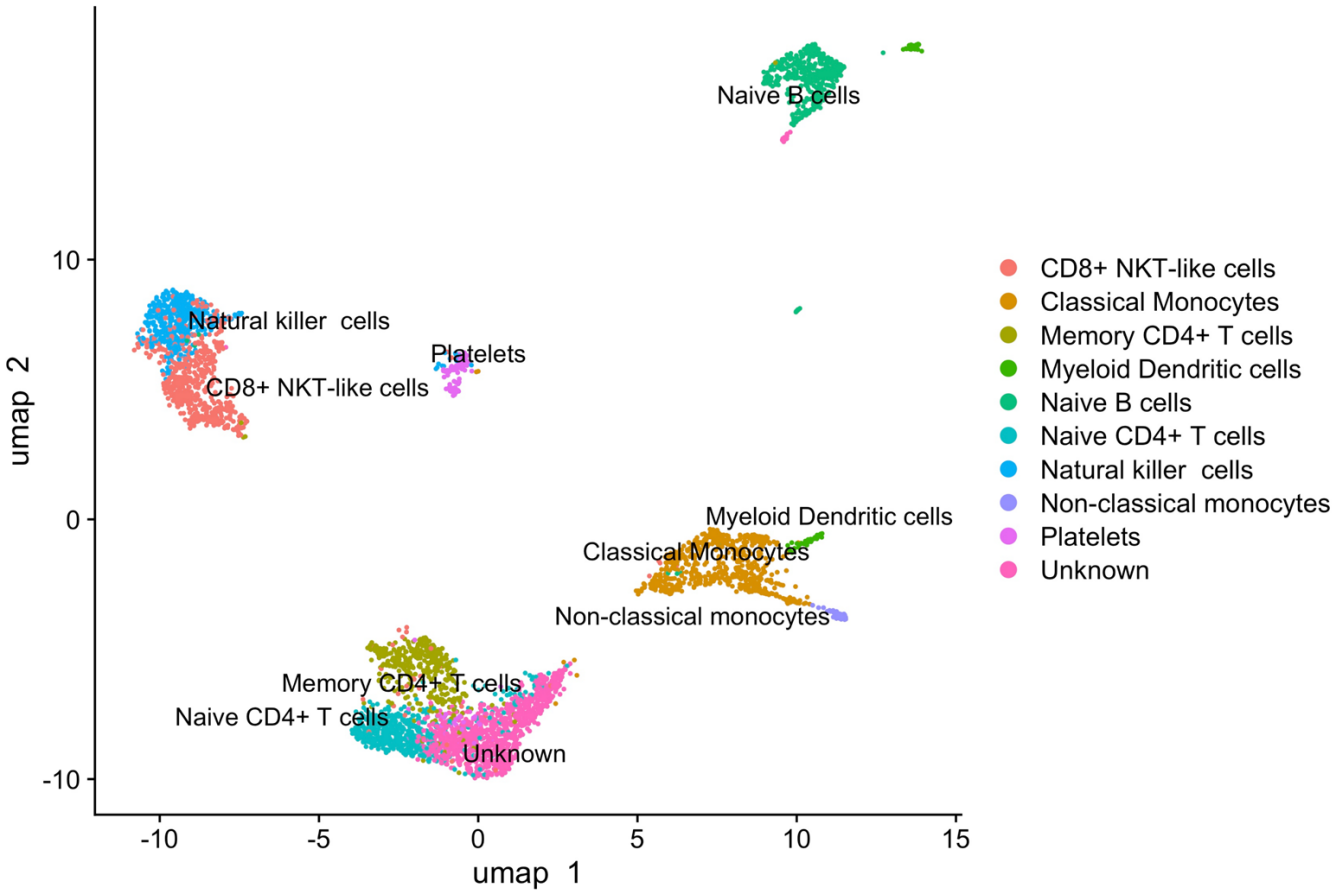
Uniform manifold approximation and projection plot of scRNAseq data. Each dot represents a single cell isolated from PBMCs samples; cells that display similar transcriptomics profiles are closer to each other than they are to any other cell. Colors indicate annotation of identified cell types.

The sequencing experiment yielded 182 108 980 reads (mean = 30 351 497; range: 15 771 427–43 849 499), and a total of 4211 cells were deemed as high‐quality after quality controls (Figures S1–S5). Cell‐type annotation was performed using the SCType Cell Marker Database for “Immune system” to identify transcriptional profiles. Overall, we identified nine cell types (Figure 1): classical monocytes, non‐classical monocytes, and myeloid dendritic cells belonging to the myeloid lineage; Natural Killer (NK) cells, CD8^+^ NK T‐like cells, memory CD4^+^ T cells, naïve CD4^+^ T cells, and naïve B cells belonging to the lymphoid lineage; and platelets. All cell types were present in both pre and post run samples. Differential expression analysis (DEA) and gene set enrichment analysis (GSEA) were conducted to identify differences in modulated transcripts and functional pathways in each cell type among pre and post run timepoints.

### 3.1 Myeloid Cells Display a Stress Response and Harbor Active Viral Processes

DEA identified 23 differentially expressed genes (20 downregulated and 3 upregulated, adjusted *p* < 0.05 and |log2FoldChange| > 0.58) in classical monocytes (Table 1): genes related to chemokines (*CCL20*, *CCL3*, *CCL3L1*, *CCL4*, *CCL4L2*, *CXCL2*, *CXCL8*), interleukin‐1β (*IL1B*), lysozyme (*LYZ*), and prostaglandins (*PTGS2*) production were downregulated in post run timepoint. Moreover, *HSPA1A* was upregulated, while *G0S2*, *IER3*, and *SOD2* were downregulated. No significantly modulated genes were detected in myeloid dendritic cells and non‐classical monocytes after correction for multiple testing.

**TABLE 1:** Log2FoldChanges of statistically significant differentially expressed genes are reported in the table for each cell type.

Gene	Classical monocytes	Memory CD4^+^ T cells	Naive B cells	Natural killer cells	Platelets	Unknown
*CCL20*	−2.42	—	—	—	—	—
*CCL3*	−1.73	—	—	—	−1.84	—
*CCL3L1*	−1.84	—	—	—	—	—
*CCL4*	−1.88	—	—	—	−1.73	—
*CCL4L2*	−1.73	—	—	—	—	—
*CDK11A*	−3.11	−2.86	−3.41	−2.73	—	−3.20
*CXCL2*	−1.12	—	—	—	—	—
*CXCL8*	−1.48	—	—	—	—	—
*DNAI1*	−3.62	—	—	−2.71	—	−2.90
*FOS*	1.07	—	—	—	—	—
*G0S2*	−1.23	—	—	—	—	—
*HLA‐DRA*	−0.85	—	—	—	—	—
*HSPA1A*	2.26	—	—	—	—	—
*IER3*	−1.53	—	—	—	—	—
*IL1B*	−0.93	—	—	—	—	—
*JUN*	—	—	—	−2.66	—	—
*LYZ*	−0.83	—	—	—	—	—
*MARCKS*	−1.16	—	—	—	—	—
*MLST8*	−1.80	—	—	—	—	—
*PIM1*	1.76	—	—	—	—	—
*PLAUR*	−1.26	—	—	—	—	—
*PTGS2*	−1.27	—	—	—	—	—
*SERPINA1*	−1.49	—	—	—	—	—
*SOD2*	−0.85	—	—	—	—	—

*Note:* “—” indicates that the modulation of the reported gene was not statistically significant for that cell type after correction for multiple testing. The pre‐marathon timepoint was used as the base level for the analysis.

**FIGURE 2:**
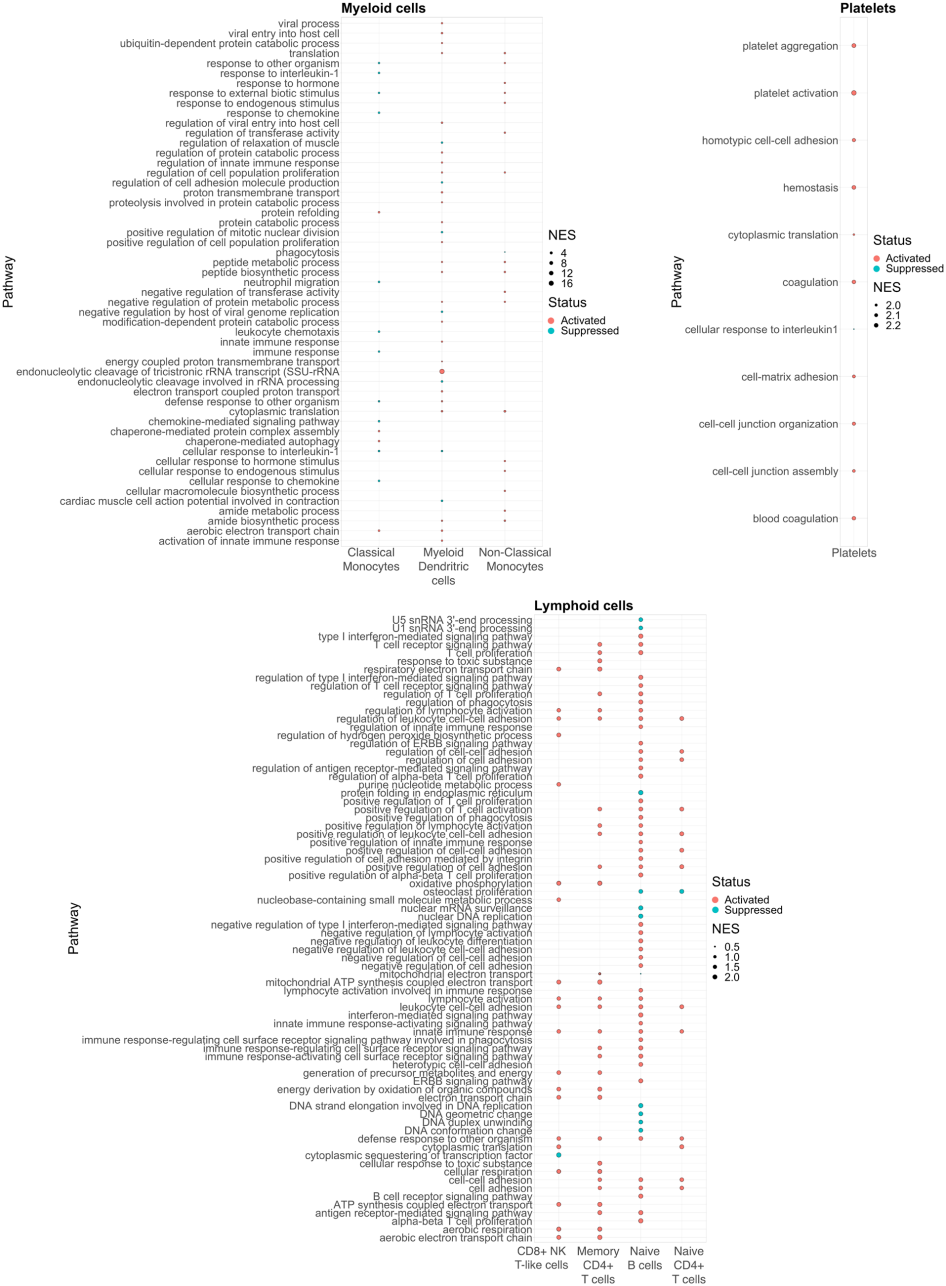
Dotplots of gene set enrichment analysis results. Statistically significant enriched pathways for each identified cell type are shown in the plots. NES, normalized enrichment score.

GSEA revealed modulated pathways (*p* < 0.05) in various cell types post run (Figure 2, Table S1). Overall, myeloid lineage cells displayed a cell type‐dependent activation of pathways related to stress response (6 related pathways, *p* < 0.04), peptide metabolism (6 related pathways, *p* < 0.02), and viral processes (4 related pathways, *p* < 0.03) post run. Protein refolding, chaperone‐mediated autophagy, and cellular respiration were activated in classical monocytes (*p* = 0.04), whereas response to chemokines was suppressed (*p* = 0.01). Non‐classical monocytes exhibited active translation and peptide biosynthesis, and suppressed phagocytosis (*p* = 0.01; 0.01 and 0.04, respectively). Finally, dendritic cells presented active viral processes (4 related pathways, *p* < 0.03), cytoplasmic translation and proton transmembrane transport, and diminished cellular response to interleukin 1 (*p* = 0.01; 0.01 and 0.04 respectively).

### 3.2 Lymphoid Cells Shift Towards Immune Activation

DEA results indicated 3 differentially expressed genes (*p* < 0.05 and |log2FoldChange| > 0.58) in cells belonging to the lymphoid lineage. Of note, *CDK11A*—which plays a role in cell cycle progression, cytokinesis, and apoptosis—was downregulated in most lymphoid lineage cell types. In addition, GSEA results indicated that lymphoid lineage cells shifted towards immune activation in all cell types (24 related pathways, *p* < 0.01; Figure 2, Table S1). Naïve CD4^+^ T‐cells displayed active proliferation, cytoplasmic translation, and leukocyte adhesion pathways (*p* = 0.02; 0.01 and 0.01 respectively); naïve B cells presented active interferon‐mediated and cell surface receptor signaling pathways (*p* = 0.001), and CD8^+^ NK T‐like cells indicated immune response activation (6 related pathways, *p* < 0.02). No significantly modulated pathways were detected in NK cells.

### 3.3 Platelets Indicate Activation of the Coagulation Process

In addition to PBMCs, we were able to isolate and analyze platelets. *CCL3* and *CCL4* genes, encoding for chemokine ligands, were found to be downregulated in platelets through DEA. Moreover, GSEA indicated the activation of platelet aggregation, cytoplasmic translation, blood coagulation and cell‐matrix adhesion (*p* = 0.005) and the suppression of response to interleukin 1 (*p* = 0.02).

### 3.4 Half‐Marathon Running Does Not Determine Cell Abundance Modulation

**FIGURE 3:**
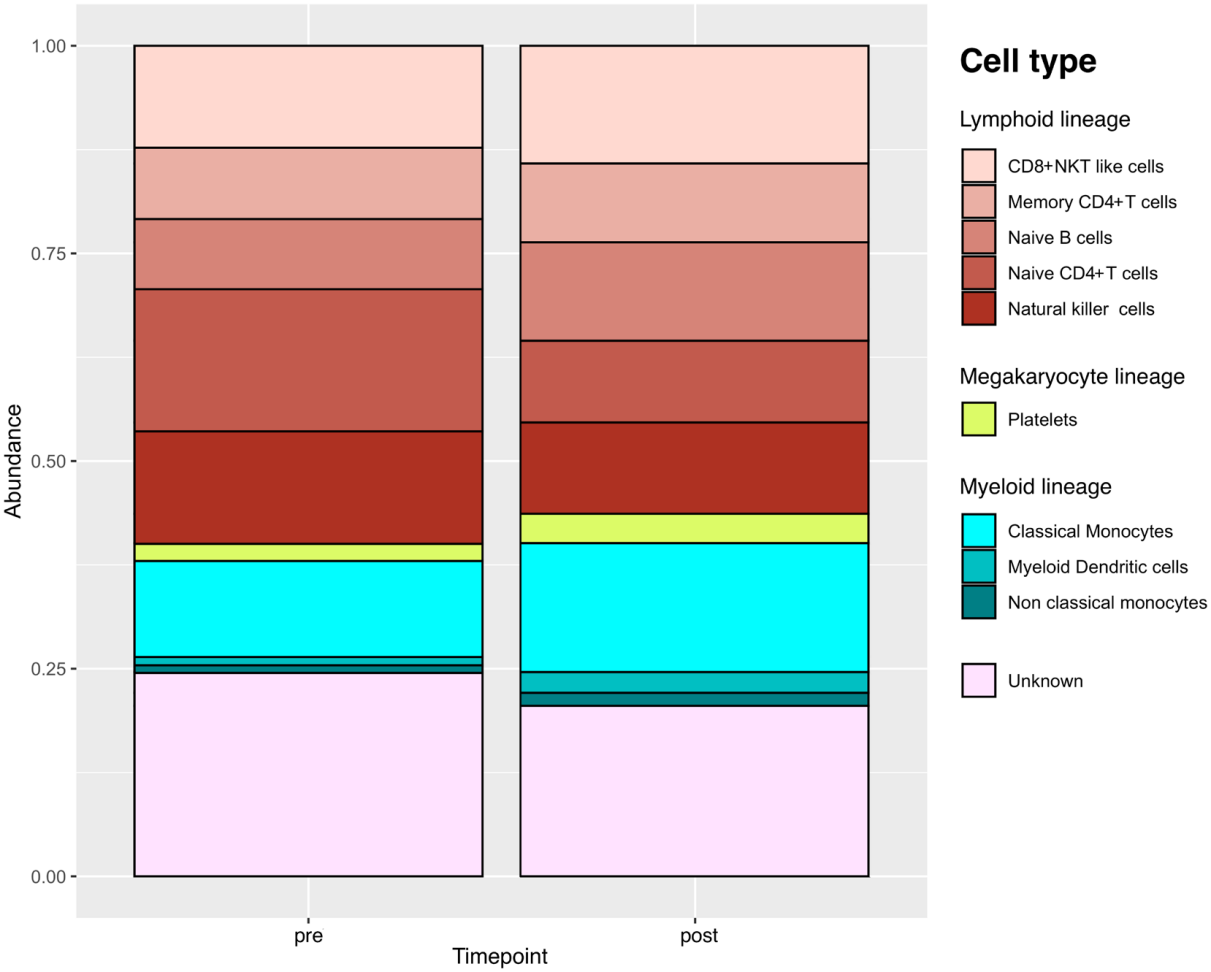
Normalized cell types composition of pre and post run samples. Timepoint pre, pre run; timepoint post: post run.

Cell type differential abundance analysis (Figure 3, Tables S2 and S3) was performed to assess whether cell types were depleted or enriched after half‐marathon running. Results indicated that no cell type displayed statistically significant changes in abundance; nonetheless, we observed a slight tendency (*p* = 0.09) towards myeloid dendritic cell enrichment after running.

### 3.5 Pseudo‐Bulk Analysis Could Offer Complementary Findings to Classical Single‐Cell Analysis Methods

Sequencing data were also analyzed simulating a bulk RNAseq experiment. DEA results indicated a total of 27 modulated genes (Table 2); 12 of them resulted to be modulated also following the single‐cell analysis approach (Table S4, which also contains a column reporting references of works that also found the same statistically significant differentially expressed genes). Notably, *CDK11A* gene was downregulated in 4 cell types (naïve B cells, memory CD4^+^ T cells, NK cells, and classical monocytes) using the single‐cell approach and showed the most significant *p*‐value using the pseudo‐bulk approach. Interestingly, 15 genes resulted as modulated only following the pseudo‐bulk analysis approach. Among them, the following genes were found to be downregulated: *CXCR4*, a mediator of bacterial lipopolysaccharide‐induced inflammatory response; *JUNB*, involved in interleukin‐1 family signaling pathways; *NKG7*, essential for cytotoxic degranulation of NK cells and CD8^+^ T cells and for the activation of CD4^+^ T cells following infection; *RGS1*, an inhibitor of B cell chemotaxis; RPS10, responsible for the synthesis of proteins in the cell; and *TNFAIP3*, an inhibitor of TNF‐mediated apoptosis. On the other hand, genes linked to platelet activation and platelet‐derived chemokine family (*PF4* and *PPBP*), mitochondrial genes linked to the electron transport chain (*MT‐CO1* and *MT‐CO2*), and heat‐shock proteins (*HSP90AA1* and *HSPA8*) were found to be upregulated.

**TABLE 2:** Log2FoldChanges of statistically significant differentially expressed genes are reported in the table. The pre‐marathon timepoint was used as the base level for the analysis.

Gene	Log2FoldChange	Adjusted *p*
*CDK11A*	−3.53	1.09E‐20
*DNAI1*	−3.52	6.77E‐18
*CCL3*	−1.57	2.51E‐06
*CCL4*	−1.44	2.51E‐06
*CXCL8*	−1.43	7.16E‐06
*CCL3L1*	−1.73	1.08E‐05
*MLST8*	−2.06	3.24E‐05
*PPBP*	1.56	1.44E‐04
*MYC*	1.91	1.44E‐04
*CCL4L2*	−1.58	1.72E‐04
*IGHA1*	1.66	7.72E‐04
*MT‐CO1*	1.45	1.13E‐03
*NKG7*	−1.10	5.07E‐03
*MT‐CO2*	1.35	5.07E‐03
*PF4*	1.36	5.89E‐03
*JUNB*	−0.96	1.11E‐02
*HSPA8*	1.10	1.12E‐02
*TNFAIP3*	−0.89	1.96E‐02
*IER3*	−1.35	2.51E‐02
*HSP90AA1*	0.90	2.72E‐02
*CXCR4*	−0.82	4.27E‐02
*RGS1*	−1.05	4.37E‐02
*IL1B*	−0.86	4.37E‐02
*CAVIN2*	1.33	4.37E‐02
*RPS10*	−0.86	4.37E‐02
*CXCL2*	−1.02	4.57E‐02
*PIM1*	1.18	4.57E‐02

GSEA revealed only two significantly downregulated pathways (Table 3): cellular response to interleukin‐1 and eosinophil chemotaxis (*p* = 0.04). The former was statistically significant in four different cell types (classical monocytes, myeloid dendritic cells, naïve B cells, and platelets), whereas the latter did not result as statistically significant using the single‐cell approach. Nonetheless, the leukocyte chemotaxis, granulocyte chemotaxis, and lymphocyte chemotaxis pathways resulted to be modulated in 3 different cell types (naïve CD4^+^ T cells, naïve B cells, and classical monocytes).

**TABLE 3:** Gene set enrichment analysis results. The pre‐marathon timepoint was used as the base level for the analysis.

ID	Description	Enrichment Score	NES	*p*	*p*‐adj	*q*
GO:0071347	Cellular response to interleukin‐1	−0.85	−2.22	2.03E‐05	0.04	0.04
GO:0048245	Eosinophil chemotaxis	−0.99	−1.92	2.08E‐05	0.04	0.04

Abbreviations: ID, gene ontology identification code; NES, normalized enriched score; *p*‐adj, adjusted *p*‐value.

## 4 Discussion

Recent research focused on the effects of practising endurance activities on the immune system, however contrasting hypotheses are still being debated. A previous study by Condeminas and colleagues [15] focused on the modulation of gene expression levels: they collected whole blood samples from non‐elite athletes before and after participating in the 2016 Barcelona Marathon and performed bulk RNA sequencing. Another recent study by Yu et al. [14] focused on the characterization of immune cell functions by carrying out a scRNAseq analysis of PBMCs after a bout of symptom‐limited cardiopulmonary exercise tests or a marathon, including only male athletes. In our work, we performed a transcriptomic analysis of PBMCs at single‐cell resolution to shed light on endurance exercise‐mediated immune modulation, taking into consideration both male and female amateur runners. Despite our study's small sample size, including female individuals could help highlight generalizable transcriptional responses attenuating the possible gender‐specific modulations.

In this work, we identified cell types belonging to the myeloid, lymphoid, and megakaryocyte lineage based on single cells transcriptional profiles. As regards the myeloid lineage, we identified a total of three cell types: classical monocytes, non‐classical monocytes, and myeloid dendritic cells. DEA results indicated that classical monocytes displayed the highest degree of transcriptional regulation. Of note, a variety of chemokine ligands (*CCL20*, *CCL3*, *CCL3L1*, *CCL4*, *CCL4L2*, *CXCL2*, *CXCL8*) were downregulated immediately after running. Among their related pathways are macrophage migration inhibitory factor‐mediated glucocorticoid regulation, which counter‐regulates glucocorticoid suppression of immune cell responses, and TGF‐β pathway. Interestingly, TGF‐β was reported to be a survival factor for monocytes, which rapidly activated apoptosis in its absence [28]. Genes related to an increased response to stress were also modulated, which could be expected after high‐intensity exercise. Moreover, *IL1B* (encoding a member of the interleukin 1 cytokine family), *LYZ* (encoding lysozyme), and *PTGS2* (encoding a key enzyme in prostaglandin biosynthesis) were found to be downregulated, suggesting a decreased inflammatory response following—or even caused by—physical exercise. We acknowledge that batch effects, particularly small but consistent biases in cell‐stress‐related gene expression, might significantly impact DEA. To address this, we have ensured transparency by including UMAP plots of each sample with and without anchoring in the supporting information (Figures S4 and S5). While we cannot exclude that modulation of immediate response genes could be a methodological artifact caused by PBMCs isolation, we found that our results are consistent with previously reported ones [14, 29]. In particular, the work by Maqueda et al. (in which no density gradient centrifugation was performed) also found *FOS*, *HSP90AA1*, *HSPA1A*, *HSPA8*, *IER3*, *JUNB* to be modulated, and the work by Yu et al. also found *JUN* to be modulated, among other genes. These results were further supported by GSEA findings, which showed suppression of immune response‐related pathways (e.g., response to chemokine, response to external biotic stimulus, leukocyte chemotaxis). No significantly single modulated genes were detected in myeloid dendritic cells and non‐classical monocytes; nonetheless, pathways analysis revealed a decreased inflammatory response in both cell types. Interestingly, active viral processes were detected in dendritic cells. This could suggest that short‐term immunosuppression takes place after half‐marathon running, generating a window of opportunity that may increase susceptibility to viral diseases. Overall, these results could suggest that the myeloid lineage cells suppress their immunological functions in favor of stress response and molecular repair. These findings support the “open window” hypothesis [14, 29, 30] and previous reports in the literature suggesting a diminished immune response after physical exercise [14, 15] and highlight the role of myeloid cells in this phenomenon. In addition, our data substantiate an activation of autophagic response in classical monocytes similar to the one observed in mesenchymal circulating progenitors [9].

Regarding the lymphoid lineage, we identified 5 cell types: NK cells, CD8^+^ NK T‐like cells, memory CD4^+^ T cells, naïve CD4^+^ T cells, and naïve B cells. DEA detected a downregulation of *CDK11A* in most lymphoid lineage cell types, suggesting an increased lymphoid cells survival after high‐intensity exercise. Additionally, GSEA results displayed activation, proliferation, and defense response to other organisms in naïve CD4^+^ T cells, CD8^+^ NK T‐like cells, and naïve B cells, indicating an active lymphoid response. These results are concordant with previous literature [31] supporting the “acute stress‐induced immunoenhancement” hypothesis [32] but offer a more detailed landscape of cell type‐specific responses. While we do not have information regarding changes in leukocyte localization, lymphoid cells active proliferation and increased adhesion could hint at a demargination to prepare the organism for potential immune challenges. Of note, memory CD4^+^ T cells showed activation of pathways related to energy generation through the electron transport chain. Since during physical exercise humans need and consume more energy compared to a resting condition, we expected such results.

In addition to PBMCs, we were able to explore platelets as proxies for megakaryocyte's response to endurance exercise. While we did not set out to explicitly capture the modulatory dynamics in platelets (they should be removed during the isolation of PBMCs), the residual platelets were equally represented in abundance across all analyzed samples and in sufficient numbers to be investigated. We recognize that this could lead to a bias in platelets abundance modulation analysis results; however, since the difference in platelets abundance was not significant in our analysis, the differential expression and pathways analyses results are not affected by possible biases linked to abundance differences. The study results indicate a decreased response to interleukin 1, and activated cell‐matrix adhesion and platelet aggregation compatible with blood coagulation. Interestingly, systemic inflammation—that could be caused either by intense exercise and/or infection—can activate and amplify coagulation [33, 34], suggesting the possibility of exercise‐induced hypercoagulability. These findings are consistent with our observations from a previous study conducted on 21 runners who participated in the 2016 edition of “Run 4 Science”. Through a proteomics study, where we compared the modulation of serum proteins after physical activity, we observed that, in addition to the activation of immune response, inflammatory response, and detoxification processes, coagulation was also modulated, particularly the coagulation factor XII [35]. The effect of a single acute exercise session on clot formation has been previously evaluated in healthy young individuals. In particular, it has been demonstrated that there is an increase in the density of clot microstructure immediately after an acute session of both low and high‐intensity cycling exercise or following a moderate‐intensity single‐leg knee extension exercise [36, 37]. Importantly, a recent study conducted on stroke patients and healthy individuals demonstrated that exercise temporarily raises the risk of blood clot formation in both groups. However, due to the higher baseline thrombogenicity in stroke patients, their risk of forming blood clots post‐exercise may be greater [38].

Moreover, we performed a cell type differential abundance analysis to assess whether cell types were depleted or enriched after half‐marathon running. Results indicated that no cell type displayed statistically significant changes in abundance, which is in contrast with a recently published work proposing a reduction of T cells immediately following exercise [14]. However, this result could be due to our study's relatively small sample size, which reduces the chance of detecting a true effect, especially when the effect size is small. In fact, while in our study we focused only on dynamics taking place immediately after half‐marathon running, the peak of cell‐type abundance modulations has been found to happen 1 h after intense exercise [14].

Finally, we explored our data following a pseudo‐bulk analysis approach (i.e., simulating a bulk RNAseq experiment) to gain further biological insights. Interestingly, some genes resulted as modulated only following the pseudo‐bulk analysis approach, indicating that this approach could offer complementary findings to classical single‐cell analysis methods. Conversely, results indicate a loss of power to detect modulated pathways when compared to a single‐cell approach. Overall, these results are concordant with the results of the single‐cell analysis approach but highlight possible additional players underlying the immune modulation observed after endurance activity.

The major limitation of our study is the small sample size, which might restrict the generalizability and reliability of the results. In particular, given the nature of the event, our sample is representative of young, trained people within a narrow age range (29 ± 5 years), and we cannot extend our results across the different stages of life. Even though we analyzed a small number of samples due to the high cost of single‐cell sequencing, the use of this sequencing method could provide a way to investigate changes in different cell populations, thus generating new hypotheses in the field. Moreover, our results are concordant with previous reports in the literature exploring immune response after physical exercise, supporting the reliability of the analysis results.

It is important to point out that our study focused only on modulatory dynamics taking place immediately after half‐marathon running. However, a previous study [14] found that more than 24 h recovery time is required to reach a baseline expression profile, thus suggesting that alterations persist for a considerable amount of time. Moreover, the observed changes could be attributable to either direct or indirect effects of performing an endurance activity. The integration of multiple omics approaches and the inclusion of more individuals in future studies will help elucidate the mechanisms underlying such dynamics.

In conclusion, performing a high‐intensity physical exercise like running a half‐marathon has a great impact on the transcriptional dynamics of immune cells. Our comprehensive transcriptomic analysis of immune cells at the single‐cell level of both male and female individuals does not support a homogeneous immune response to half‐marathon running but rather depicts complex dynamics attributable to different actors. On one hand, the lymphoid lineage supports the “acute stress‐induced immunoenhancement” hypothesis by shifting towards immune activation; on the other, myeloid cells display a stress response and harbor active viral processes, supporting the “open window” hypothesis. Moreover, platelets activate the coagulation process after half‐marathon running. If our findings were to be confirmed in a larger cohort, lines of action could be evaluated to prevent possible harmful effects linked to exercise‐induced hypercoagulability and the activation of inflammatory mechanisms. Such insights and further studies contribute to the comprehension of the interplay between exercise and health and may help establish future training routines or nutritional guidelines based on individual gene expression levels.

## 5 Perspective

The findings presented in this study contribute to the ongoing research surrounding the effects of endurance exercise on the immune system, addressing the contrasting hypotheses of short‐term immunosuppression versus acute stress‐induced immunoenhancement. Previous research, such as the study by Condeminas et al. [15], has examined gene expression modulation in non‐elite athletes participating in marathons, while Yu and colleagues [14] focused on immune cell function characterization following exercise. Building upon this, our study provides a comprehensive transcriptomic analysis of PBMCs at the single‐cell level, taking into consideration both male and female amateur runners. Despite the small sample size of our study, the inclusion of female individuals could help in highlighting generalizable transcriptional responses attenuating the possible gender‐specific modulations. By elucidating the transcriptional dynamics of immune cells post half‐marathon, our findings offer valuable insights into endurance exercise‐mediated immune modulation, with potential implications for optimizing training strategies and enhancing athletic performance while minimizing health risks. This research highlights the importance of considering individual responses to exercise in the development of personalized training regimens.

## Author Contributions

Conceptualization: Giovanni Malerba. Methodology: Laura Veschetti, Cristina Patuzzo, Giovanni Malerba. Software: Laura Veschetti. Validation: Laura Veschetti, Cristina Patuzzo. Formal analysis: Laura Veschetti. Investigation: Cristina Patuzzo, Michela Deiana, Samuele Cheri, Francesca Griggio. Resources: Giuseppe Lippi, Federico Schena, Luca Dalle Carbonare, Cantor Tarperi, Maria Teresa Valenti, Giovanni Malerba. Data Curation: Cristina Patuzzo, Laura Veschetti. Writing – original draft: Laura Veschetti, Cristina Patuzzo, Mirko Treccani. Writing – review and editing: Lucas Moron Dalla Tor, Michela Deiana, Francesca Griggio, Giuseppe Lippi, Federico Schena, Daniele De Santis, Cantor Tarperi, Elisabetta Trabetti, Maria Teresa Valenti, Giovanni Malerba. Visualization: Laura Veschetti. Supervision: Giovanni Malerba. Funding acquisition: Elisabetta Trabetti, Giovanni Malerba.

## Consent

Written informed consent was obtained from all participants.

## Conflicts of Interest

The authors declare no conflicts of interest.

## Ethics Statement

The study was approved by the ethical committee of Azienda Ospedaliera Universitaria Integrata of Verona, Italy (no. 1538; Dec. 3, 2012; local ethical committee of Azienda Ospedaliera Integrata di Verona) and was conducted in accordance with the Declaration of Helsinki.

## Supporting information


Figure S1.

Table S1.

## Data Availability

The data that support the findings of this study are openly available in European Nucleotide Archive at https://www.ebi.ac.uk/ena/browser/home. Sequencing data were submitted to the European Nucleotide Archive database with project no. PRJEB76303.
