# Supplementary material for: Single‐Cell RNA Sequencing Analysis Reveals Exercise‐Induced Transcriptional Dynamics in Half‐Marathon Runners
Source: Scand J Med Sci Sports. 2025 Jan 16;35(1):e70018. doi: 10.1111/sms.70018 (PMC11737006; doi:10.1111/sms.70018)
Supplement: Supplementary file 1 — Figure S1. [file SMS-35-e70018-s002.docx]

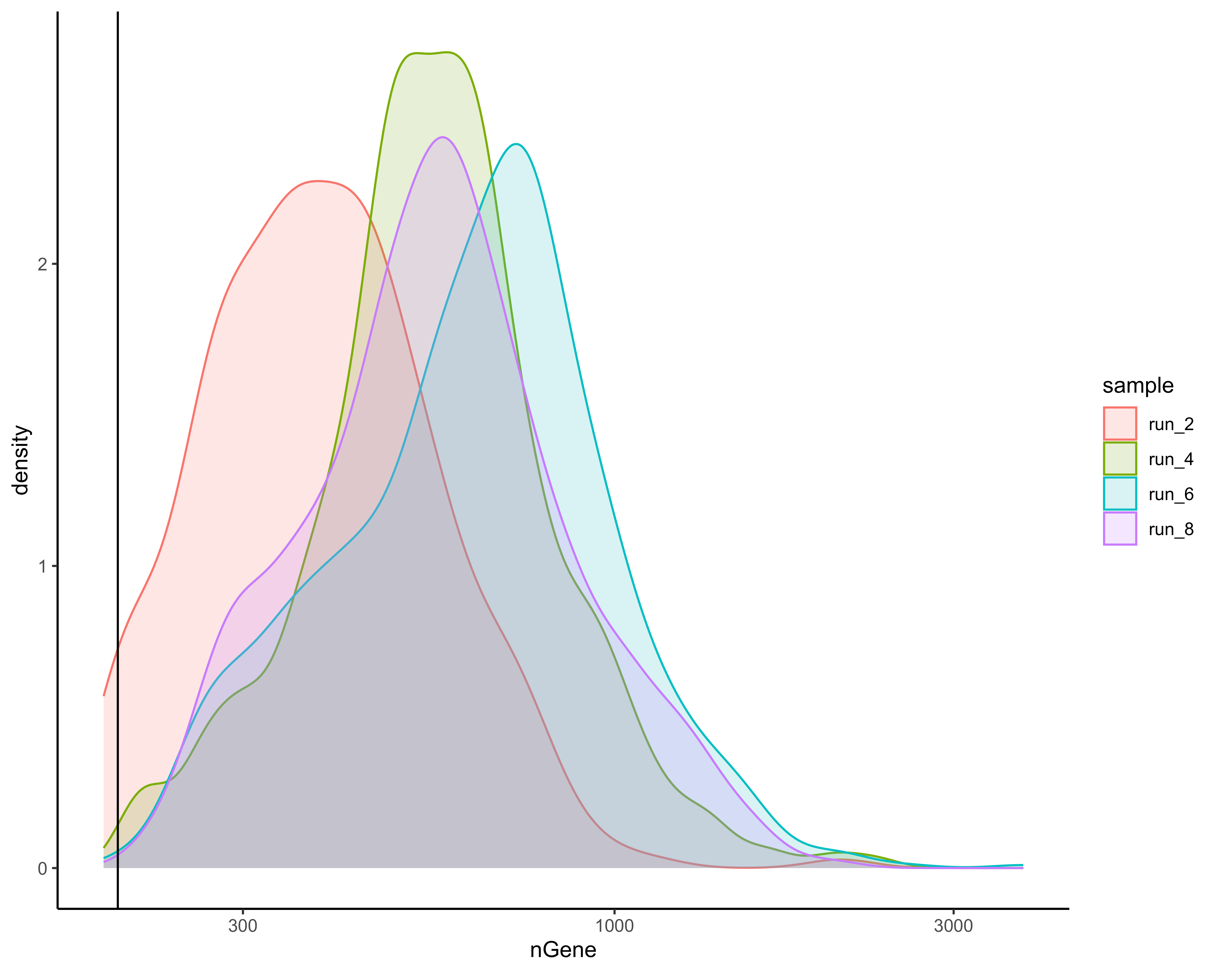


**Figure S1** Density plot of number of genes detected in each sample.


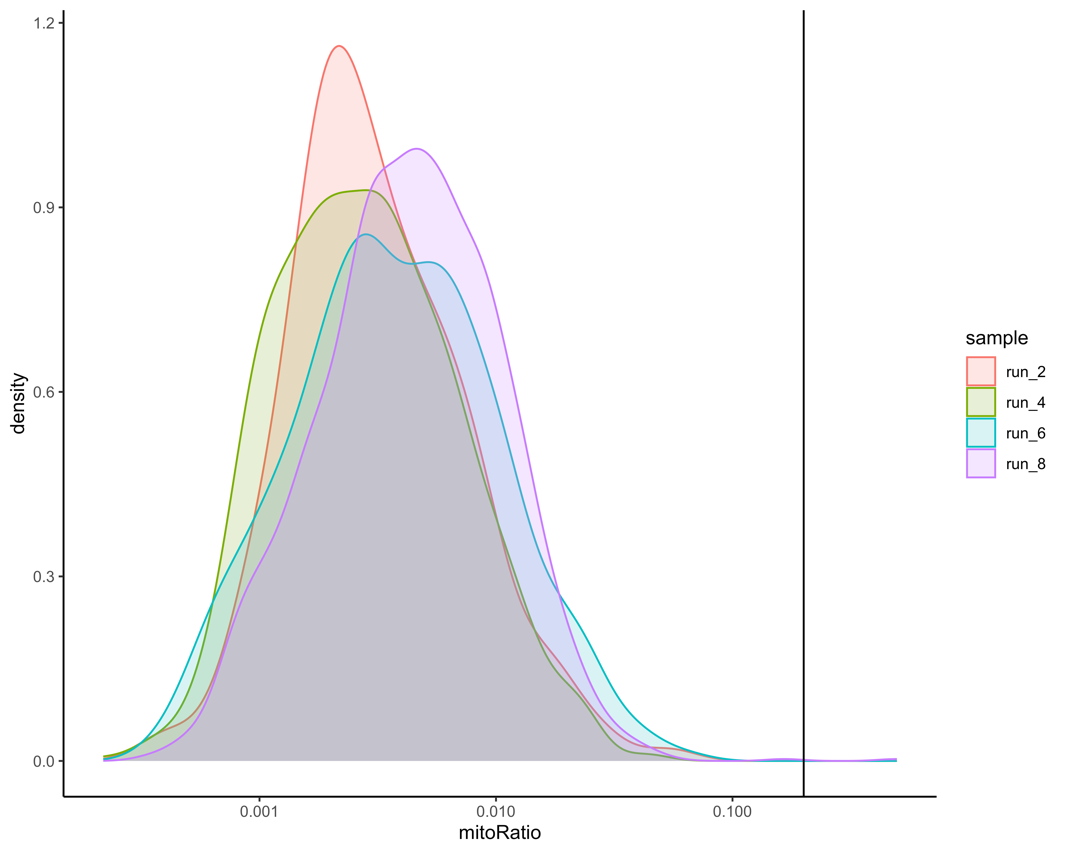


**Figure S2** Density plot of number of mitochondrial content detected in each sample.


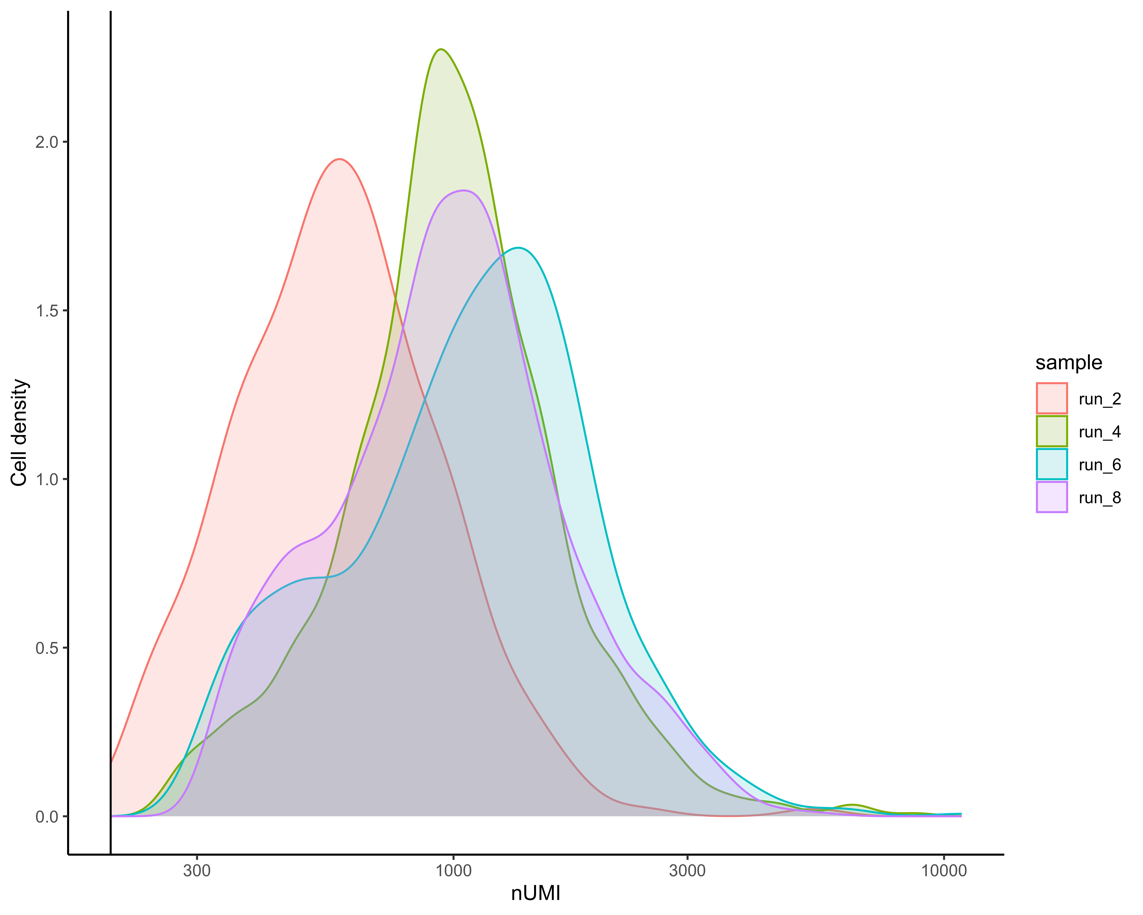


**Figure S3** Density plot of number of universal molecular identifiers (UMIs) detected in each sample.


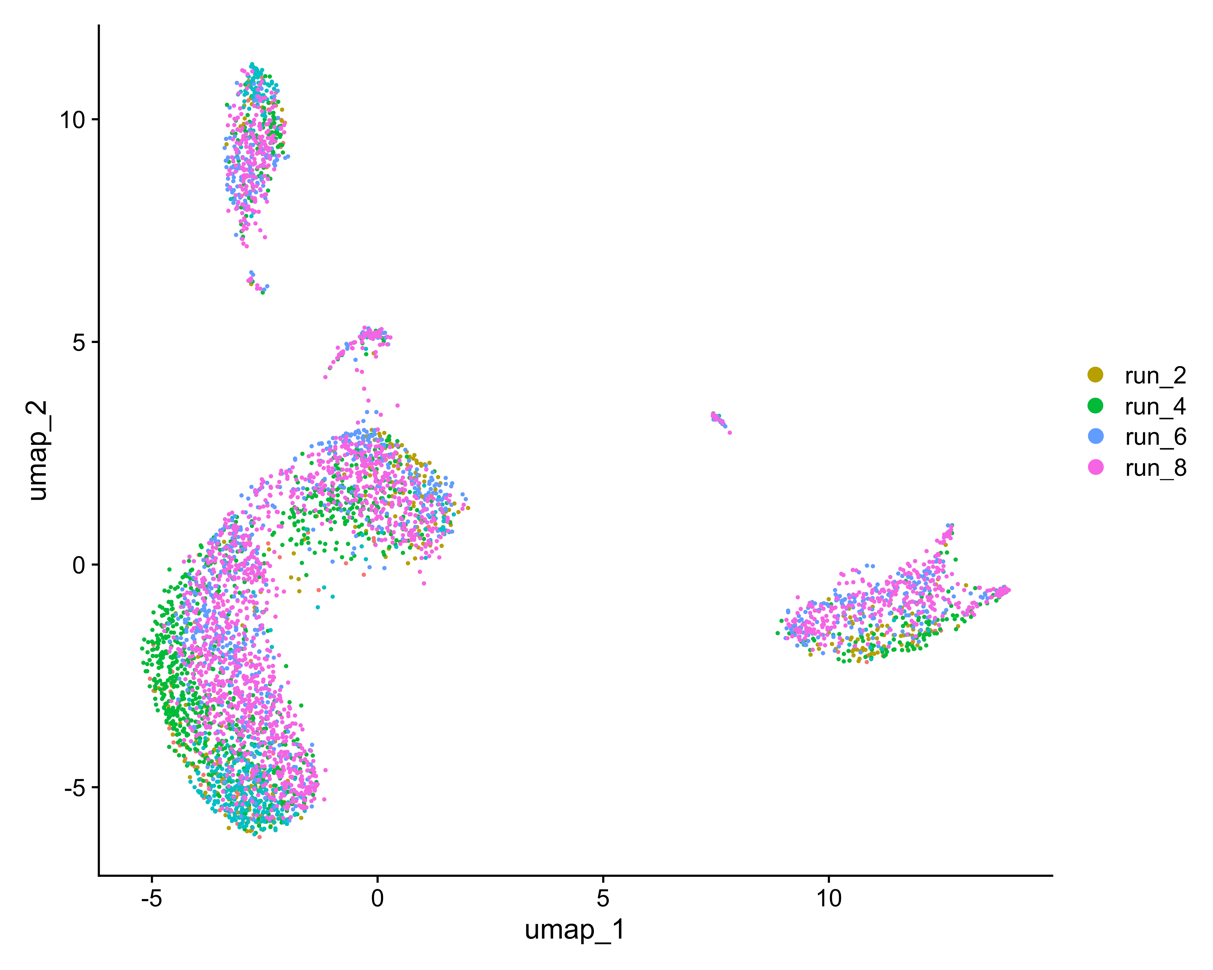


**Figure S4** Uniform manifold approximation and projection plot of scRNAseq data prior to batch effect correction. Each dot represents a single cell isolated from PBMCs samples, cells that display similar transcriptomics profiles are closer to each other than they are to any other cell. Colours indicate samples.


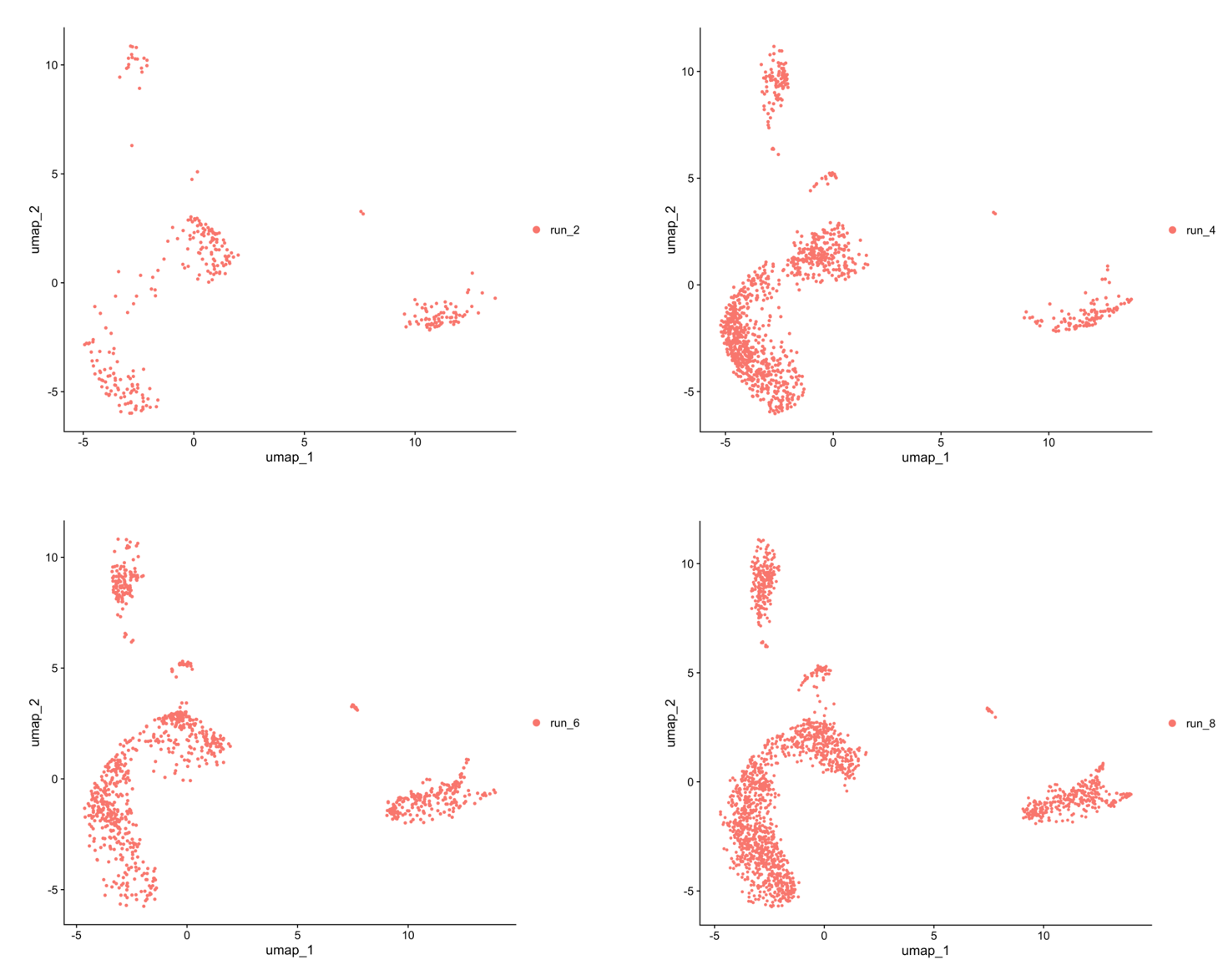


**Figure S5** Uniform manifold approximation and projection plot of scRNAseq data prior to batch effect correction. Each dot represents a single cell isolated from PBMCs samples, cells that display similar transcriptomics profiles are closer to each other than they are to any other cell. Each panel represent the samples indicated in the respective legend.
